# Modeling interaction networks between host, diet, and bacteria predicts obesogenesis in a mouse model

**DOI:** 10.3389/fmolb.2022.1059094

**Published:** 2022-11-15

**Authors:** Peter E. Larsen, Yang Dai

**Affiliations:** ^1^ Loyola Genomics Facility, Loyola University at Chicago Health Science Campus, Maywood, IL, United States; ^2^ Department of Biomedical Engineering, University of Illinois at Chicago, Chicago, IL, United States

**Keywords:** network biology, microbiome, metabolome, mouse model, computational modeling, obesity

## Abstract

Host-microbiome interactions are known to have substantial effects on human health, but the diversity of the human microbiome makes it difficult to definitively attribute specific microbiome features to a host phenotype. One approach to overcoming this challenge is to use animal models of host-microbiome interaction, but it must be determined that relevant aspects of host-microbiome interactions are reflected in the animal model. One such experimental validation is an experiment by Ridura et al. In that experiment, transplanting a microbiome from a human into a mouse also conferred the human donor’s obesity phenotype. We have aggregated a collection of previously published host-microbiome mouse-model experiments and combined it with thousands of sequenced and annotated bacterial genomes and metametabolomic pathways. Three computational models were generated, each model reflecting an aspect of host-microbiome interactions: 1) Predict the change in microbiome community structure in response to host diet using a community interaction network, 2) Predict metagenomic data from microbiome community structure, and 3) Predict host obesogenesis from modeled microbiome metagenomic data. These computationally validated models were combined into an integrated model of host-microbiome-diet interactions and used to replicate the Ridura experiment *in silico*. The results of the computational models indicate that network-based models are significantly more predictive than similar but non-network-based models. Network-based models also provide additional insight into the molecular mechanisms of host-microbiome interaction by highlighting metabolites and metabolic pathways proposed to be associated with microbiome-based obesogenesis. While the models generated in this study are likely too specific to the animal models and experimental conditions used to train our models to be of general utility in a broader understanding of obesogenesis, the approach detailed here is expected to be a powerful tool of investigating multiple types of host-microbiome interactions.

## 1 Introduction

All of us live in an intimate association with communities of microorganisms, which are collectively termed our microbiome. These microbial communities are with us our entire lives, inoculated in as at the moment of our birth, helping to guide our development, tuning our immune systems, and beginning the process of decomposition at the moment of our death. In between times, microbiomes have been implicated in a wide variety of conditions, such as IBS, inflammation, autoimmune disorders, susceptibility to certain cancers, and depression (Huttenhower et al., 2012; Wylie et al., 2012; Putignani et al., 2014; Yang et al., 2016a; Schmidt et al., 2018). The gut microbiome is also known to play a role in obesity (David et al., 2014; Bianchi et al., 2018; Racz et al., 2018; Tseng and Wu, 2018). Obesity results in a significantly increased risk in mortality (Global et al., 2016), and is associated with elevated risk for serious health conditions such as hypertension, type two diabetes, heart disease, stroke, osteoarthritis, inflammation, and some cancers (Consortia. Clinical, 1998; Kasen et al., 2007; Bhaskaran et al., 2014; Ryan and Heaner, 2014; Blander et al., 2017). The CDC estimates that the cost of obesity to the United States healthcare system is about $173 billion a year (Promotion, 2022). Although the microbiome is only one factor of many contributing to obesity (Rabot et al., 2016; Xiao et al., 2017), even a small decrease in the prevalence of obesity in the world’s population will lead to significant reductions in medical costs and prevent tens of thousands of premature deaths.

With such a broad range of effects on host health, it would seem that the microbiome is a profitable target for addressing diseases such as obesity. A significant complication to developing microbiome-based diagnostics or treatments is the inherent, tremendous variability in human microbiome communities, both from individual to individual and within a single individual over time (Schloissnig et al., 2013; David et al., 2014; Gerber, 2014; Putignani et al., 2014). This diversity in microbiome communities makes it difficult to attribute particular microbiome community features with a specific disease, such as obesity (Walters et al., 2014). Evidence suggests that, for many conditions, host-microbiome interactions is not due to the simple presence, absence, or relative abundance of any single bacterial species or taxa in the microbiome, but rather is the consequence of a network of interactions in the microbiome community (Larsen and Dai, 2015a) (Walters et al., 2014; Larsen and Dai, 2015b). Therefore, to understand host-microbiome interactions, a network-based approach is required.

One way to overcome the challenge of microbiome diversity to address how the microbiome predisposes a host to obesity is to turn to experimental animal models. The use of a mouse model has many significant advantages (Turnbaugh et al., 2009; Bouskill et al., 2012). In particular, the microbiome of laboratory-reared mice can be rigorously controlled and standardized (Ward and Trexler, 1958). It is important, however, to determine how well and under what conditions an animal microbiome model might profitably represent a human host. Ridaura et al. (2013) describes an experiment in which the obesity phenotype of human donors is transferred into mice *via* a microbiome transplant. In this study, germ-free mice were inoculated with microbiome communities collected from twins discordant for obesity. Not only were human microbiome transplants found to be persistent in their new mouse hosts, but mice that received an “Obese” microbiome gained more weight, even on a low fat, low sugar diet, than mice that received a “Lean” microbiome transplant. So, for some aspects of human obesity, a mouse model is a valuable tool (To the best of our knowledge, the reverse experiment, transplanting a mouse into a human host, has unfortunately not yet been performed).

In this study, our goal is to leverage network-based predictive computational modeling approaches to identify mechanisms of interaction in microbiome-associated host obesity. This approach is divided into three sub-goals: 1) Predict the change in microbiome community structure in response to host diet, 2) Predict a subset of the microbiome’s metagenome, those genes associated with metabolism, from microbiome community structure, and 3) use predicted metagenomic data to model microbiome community metabolome and predict a microbiome’s obesogenesis, the likelihood that the microbiome predisposes the host to obesity. We show that network-based approaches are better predictors of host-microbiome interaction than very similar methods that do not consider networks of biological interactions. Models were trained on microbiome data collected from a variety of previously published studies, as well as the aggregated information from thousands of sequenced and annotated bacterial genomes and the collected information for bacterial metabolic networks. Our criteria for the success of these models are 1) models can accurately predict the experiments on which models were trained, and 2) the integrated models can predict the results for biological experiments outside of the training data, highlighting the predictive abilities of network-based host-microbiome interaction models.

While the specific network-based models generated in these analyses are likely too narrowly focused to be of general utility in the study of microbiome-associated obesogenesis, we propose that the broader approach presented here, integrating multiple individually trained models into a predictive system, can be applied to a range of host-microbiome interactions and will be a valuable tool for future studies.

## 2 Materials and methods

### 2.1 Collected datasets

For this study, a collection of previously published mouse microbiome studies, each investigating some aspect of host-microbiome interactions, were leveraged for our models.

#### 2.1.1 Effect of diet on microbiome community

In the manuscript by Carmody Rachel et al. (2015), authors report the changes in microbiome community structure in response to changes in host diet in an experimental mouse model system. Diets were provided as a gradient from High Fat (HF) to Low Fat (LF) diet conditions. Adult male C57BL/6J mice raised on LF diets were fed mixed LF and HF-diet pellets in proportions of 0, 1, 10, 25, 50, 75, or 100% HF diet for 7 days. Data were collected from 33 mice from the initial microbiome communities and again after 7 days on the new diet for a total of 66 microbiome community observations. Data from this experiment were collected from the metagenomics database MG-RAST (http://metagenomics.anl.gov/) using the “MGRASTer” tool (https://github.com/braithwaite/MGRASTer/) in R (https://www.r-project.org/).

#### 2.1.2 Metagenomic diversity of mouse microbiomes

A study of the diversity of mouse microbiomes by Xiao et al. (2015) is comprised of 184 mouse gut microbiomes with paired microbiome community structure and metagenome sequence data. Microbiomes were collected from eight mouse strains that were maintained at seven different housing labs/facilities. 68% of the mice in the dataset are male and 74% were raised on a LF diet. There were 1558 unique enzyme functions (using Enzyme Commission (EC) ontology annotations) present in the available metagenomic data that make up the enzyme function profiles of a microbiome community. Microbiome community structure and metagenomic data are available through the GigaDatabase website (http://gigadb.org/dataset/100114).

#### 2.1.3 Obesity and microbiome

A key complication in the studies of the microbiome’s effect on obesity is to distinguish the causal interactions of diet on the microbiome from the effects of the microbiome on obesity. In a study published by Xiao et al. (2017), this challenge is addressed by considering two different mouse genotypes. In mouse strain C57BL/6J, treatment of mice with a COX-inhibitor prevents HF-diet induced obesity. A total of 30 Sv129 mice (10 LF diet, 10 HF diet, and 10 HF + inhibitor) and 24 BL6 mice (7 LF diet, 8 HF diet, and 9 HF + inhibitor) were used in this study. The microbiome community structure data from this experiment are available through the GigaDatabase website (http://gigadb.org/dataset/100271).

#### 2.1.4 Microbiome transplant

The manuscript by Ridaura et al. (2013), as previously mentioned in the Introduction, describes an experiment in which the obesity phenotype of human donors is transferred into mice *via* a microbiome transplant. Microbiome community structure data for “Lean” and “Obese” human microbiome transplants were collected from the Supplemental Files of the manuscript.

#### 2.1.5 Harmonizing microbiome experiment datasets

In order to integrate the selected diverse published datasets into a single computational framework, all microbiome communities need to be described using a common set of bacterial taxonomic identifiers. Using the most abundant taxa that describe communities across all microbiome datasets, twenty taxa (4 Orders, 15 Genera, and a category for “Other”) were selected (Table 1). On average, the class “Other” comprises about 16% of bacterial abundances in selected microbiome community structures. All community datasets were normalized such that total bacterial abundance sums to 100. The complete set of microbiome community datasets is available in Supplementary Data Sheet S1.

**TABLE 1:** Taxa used in mouse microbiome community structures.

Order	Genus	Description*
Actinobacteria	Collinsella	The abundance of Collinsella correlate strongly with high levels of inflammatory compounds
Bacteroidales	*Bacteroides*	*Bacteroides* species commonly found in the human gut, where they play a fundamental role in processing of complex carbohydrates
	Parabacteroides	Parabacteroides help digest high-fiber diets and their levels are elevated in the presence of resistant starches
Bacteroidetes	Porphyromonas	Porphyromonas are Gram-negative obligate anaerobes. Some species are associated with autoimmune diseases
	Anaerostipes	Anaerostipes is anaerobic, Gram-positive, and occurs in the human gut
Clostridiales	Blautia	Blautia are common in the human gut microbiome and produce acetate. IBS patients have increased levels of Blautia species
	Butyrivibrio	Butyrivibrio are common in the gastrointestinal systems of many plant-eating animals
	*Clostridium*	*Clostridium* are Gram-positive bacteria, and includes the diarrhea-causing *Clostridium difficile*
	Eubacterium	Eubacterium are common in the gut microbiome and help to digest resistant starches
	Lachnospiraceae	The Lachnospiraceae are an anaerobic bacteria found in the human gut. Members of this family are linked to obesity and may protect against colon cancer in humans by producing butyric acid
	Oribacterium	Oribacterium are found in higher abundance in the gut microbiome with high-fat diets and are potentially linked to inflammation
	Ruminococcaceae	Ruminococcaceae are common bacteria in the gut microbiome and help to digest resistant starches. Ruminococcaceae increase in abundance with a diet high in plant starches
	Ruminococcus	Ruminococcus are Gram-positive gut anaerobes commonly found in gut microbiome. They help digest resistance starches and are associated with reduced risk of diabetes and colon cancer
	*Other*	Clostridia are obligate anaerobes. They are commonly found in animal microbiomes and some can be pathogens
Erysipelotrichia	Erysipelotrichaceae	Erysipelotrichaceae increase abundance with a high-fat diet and are associated with inflammation-related disorders of the gastrointestinal tract
Lactobacillales	*Lactobacillus*	*Lactobacillus* are Gram-positive, facultative anaerobes or microaerophilic and are commonly found in the gut microbiome
Atopobium	*Any*	Atopobium are Gram-positive anaerobes
Desulfotomaculum	*Any*	Desulfotomaculum are sulfate-reducing, obligate anaerobes. Desulfotomaculum can cause food spoilage in poorly processed canned foods
Lactococcus	*Any*	Lactococcus produce lactic acid as the sole product of glucose fermentation
*OTHER*	*N/A*	

*Descriptions of taxa are collected and summarized from the NCBI taxonomy browser, (https://www.ncbi.nlm.nih.gov/Taxonomy/Browser/wwwtax.cgi).

A method for describing host diets that can accommodate arbitrary combinations of HF and LF diets is required for host-microbiome-diet interaction models. For this, host diet is described as a vector of nutrient parameters. The LF diet parameters were collected from available data sheets for ENVIGO “Teklad Custom Diet” (http://www.envigo.com/products-services/teklad/laboratory-animal-diets/), comprised of Diet Mix TD.08811 made with Mineral Mix TD.94046 and Vitamin Mix TD.94047. High Fat (HF) data parameters were collected from available datasheets for LabDiet “JL Rat and Mouse/Auto 6F” (http://www.labdiet.com/). The amino acid composition for casein in LF diet was inferred from an analysis found in (Gordon et al., 1949). There are 48 total diet parameter features, comprised of 17 protein/amino acids, 7 carbohydrates, 4 fats, 8 minerals, 11 vitamins, and Kcal/g (Table 2). For use in models, all diet parameters were defined as arbitrary values between 0 and 100. Diet parameters for high fat and low fat diets from collected experiments were normalized to values between 20 and 80 (high and low values were selected so that the model is hypothetically capable of considering diet conditions with lower or higher nutrient concentrations than those used to train the model) and log_2_ transformed.

**TABLE 2:** Mouse diet compositions for High Fat (HF) and Low Fat (LF).

	Nutrient (g/Kg)	HF	LF		Nutrient (g/Kg)	HF	LF
	Protein	19.3	18	Minerals	Calcium	1.5351	1.17
Amino Acids	Ile	0.89	0.87		Potassium	1.347534	0.66
	Leu	1.73	1.52		Magnesium	0.10449	0.22
	Lys	1.51	0.97		Iron	0.026058	0.038
	Met+Cys	0.62	0.98		Zinc	0.007095	0.0085
	Phe+Tyr	1.97	1.41		Magnesium	0.002709	0.016
	Thr	0.78	0.68		Copper	0.001333	0.0011
	Val	1.09	0.9		Iodine	0.000043	0.00021
	Trp	0.20	0.23	Vitamins	Niacin	0.0057	0.009
	His	0.49	0.44		Panthothenate	0.00304	0.0037
	Ala	0.53	1.13		Pyridoxine	0.00133	0.001
	Arg	0.65	1.03		Riboflavin	0.00114	0.0009
	Asp	1.47	1.87		Folic acid	0.00038	0.00019
	Glu	4.25	4.52		Biotin	0.000038	0.00003
	Gly	0.33	0.94		Vit B12	0.00475	0.005
	Pro	1.81	1.53		Vit E	0.0285	0.0045
	Ser	1.08	0.98		Vit A	0.00152	0.002
Carbohydrates	Carbohydrate	50.34	39.79		Vit D3	0.00038	0.00043
	Starch	11.7	38.9		Vit K	0.000143	0.002
	Glucose	0	0.12		Kcal/g	4.7	3.17
	Fructose	0	0.15				
	Sucrose	34.84	0.62				
	Lactose	3.8	0				
Fats	Fiber (cellulose)	5	15				
	Total Fat	23.2	6.2				
	Saturated	14.15	1.24				
	Mono saturated	7.192	1.37				
	Poly unsaturated	1.856	0.24				

### 2.2 Predict change in microbiome community structure in response to host diet

For this study, we leveraged the previously published Microbiome Assemblage Prediction (MAP) model approach for predicting microbiome community structure as a function of environmental data and using a learned network of microbiome community interactions (Larsen et al., 2011). Briefly described, a MAP model consists of two steps. The first is to generate a community interaction network from collected microbiome data as a directed acyclic graph, such that nodes are environmental conditions or bacterial taxa, all root nodes are environmental conditions, and interactions in the network are predicted causal interactions between environmental parameters and bacteria taxa, or between bacterial taxa. The second step is to describe the community interaction network as a system of equations, such that the abundance of a bacterial taxa can be described as a function of the values of parent nodes (environmental parameter or bacterial taxa) in the interaction network. Here, we substituted host diet parameters for “environmental conditions” in the MAP model. The data from “Effect of Diet on Microbiome Community” was used in this analysis.

While there are other tools available for predicting metagenomic data from microbiome community structure [e.g., PiCRUST (Langille et al., 2013), Tax4Fun (Asshauer et al., 2015; Scott et al., 2015)] these alternative tools use OTU-level microbiome data as input. Microbiome community data considered here was collected at a higher level of taxonomy, necessitating an alternative approach.

The network of community interactions between initial microbiome community was generated as a Bayesian Interaction Network, such that root nodes of the directed acyclic network were diet parameters and initial microbiome community, using BANJO (https://users.cs.duke.edu/∼amink/software/banjo/). BANJO was run using the parameters for “Greedy” searcher, and with a maximum of five parents per node.

The resulting microbial interaction network can be represented as the following regression model:

$${taxon}_{i}^{t}=\left(\overset{{CIN}_{i}^{diet}}{\underset{j=1}{\sum }}w_{j,i}{diet}_{j}^{t}\right)+\left(\overset{{CIN}_{i}^{{taxa}^{t}}}{\underset{k=1}{\sum }}\;w_{k,i}{taxon}_{k}^{t}\right)+\left(\overset{{CIN}_{i}^{{taxa}^{t-1}}}{\underset{l=1}{\sum }}{\;w}_{l,i}{taxon}_{l}^{t-1}\right)+c_{i}$$ (1)

Where ${taxon}_{i}^{t}$ is the relative abundance of taxon *i* at time *t*. ${CIN}_{i}^{diet}$ is the set of diet parameters that are parents of taxon *i* in microbial interaction network, ${CIN}_{i}^{{taxa}^{t}}$ is the set of taxa abundances that are parents of taxon *i* in microbial interaction network at time point *t*, and ${CIN}_{i}^{{taxa}^{t-1}}$ is the set of taxa abundances from previous time point, *t-*1, that are parents of taxon *i* in microbial interaction network. $w_{j,i}$ , $w_{k,i}$ , and $w_{l,i}$ are weights between taxon *i* and diet or taxon nodes at time *t*, or taxon nodes as time *t-1* in the microbial interaction network respectively. For interactions between taxa at time t, for $w_{k,i}$ when *k* = *i*, $w_{k,i}$ is defined as 0 (i.e. a taxon is not permitted to influence its own abundance at time t).

As a control, we also considered a non-networked model. For our non-networked model, the abundance of a microbial taxa after diet change is computed as the following regression model:

$${taxon}_{i}^{t}=\overset{A{LL}^{{diet}^{t}}}{\underset{j=1}{\sum }}w_{j,i}{diet}_{j}^{t}+\overset{A{LL}^{{taxa}^{t-1}}}{\underset{l=1}{\sum }}w_{l,i}{taxon}_{l}^{t-1}+c_{i}$$ (2)

Where $A{LL}^{{diet}^{t}}$ and $A{LL}^{{taxa}^{t-1}}$ indicate that all nutrient parameters and taxa relative abundances, not just those that are parent nodes in the microbiome interaction network, are considered as parameters in these equations.

The *w* parameters of the above regression models for microbiome community were solved using least squares estimate (QR decomposition of matrix in R). Predictive performance of the models was determined by the Pearson Correlation Coefficient (PCC) between the predicted and observed microbiome community structures (as $\log_{}^{2}$ relative abundances) where each community structure is comprised of 20 taxa. For calculating the edge weights for MAP-models, the dataset was repeatedly divided into five non-overlapping training (80%) and testing (20%) datasets.

### 2.3 Predict enzyme function profile from microbiome community structure

We have used a previously published approach for predicting the relative abundance of genes for metabolic enzymes, the microbiome’s Enzyme Function Profile (EFP), from microbiome community data, that is, presented as the relative abundance of bacterial taxa (Larsen et al., 2015a; Larsen and Dai, 2015b). Briefly, the EPF for a microbiome community can be estimated by:

$${EC}_{i}^{n}=\overset{Taxa}{\underset{j=1}{\sum }}{AveEC}_{i}^{j}\times {Taxon}_{j}^{n}$$ (3)

Where ${EC}_{i}^{n}$ is the abundance of enzyme function *i* in microbiome *n*, ***Taxa*** is the set of bacterial taxa that comprise in the microbiome community structure, ${AveEC}_{i}^{j}$ is the average number of genes for enzyme function *i* in taxon *j*, and ${Taxon}_{j}^{n}$ is the relative abundance of taxa *j* in microbiome *n*.

The data from “Metagenomic Diversity of Mouse Microbiomes” provides an opportunity to improve on this approach by optimizing equations to fit observed metagenomic data. To optimize EFP-prediction for mouse gut microbiomes, an additional term to equation was added (Yang et al., 2016a):

$${EC}_{i}^{n}=\overset{Taxa}{\underset{j=1}{\sum }}\left({AveEC}_{i}^{j}+e_{j}^{i}\right)*{Taxon}_{j}^{n}$$ (4)

Where $e_{j}^{i}$ is a constant that is added to the average enzyme function abundance for enzyme activity *i* in bacterial taxon *j*. This error term is calculated such that the range of possible values is determined from the observed standard deviation (SD) in published genomes for the number of genes for enzyme function *i* in taxon *j*.

We applied a Stochastic Hill Climbing algorithm approach and the “Metagenomic Diversity of Mouse Microbiomes” dataset to determine the values of $e_{j}^{i}\;so\;that$ the prediction of EFP is optimized for mouse microbiomes. Stochastic Hill Climbing is an iterative local search optimization method which chooses randomly from the set of all possible moves to reduce the possibility of achieving a local maximum (Russell et al., 2010; Wu et al., 2011). The selection of random taxon and random enzyme function follows a Boltzmann distribution, such that observations with higher SDs are selected more frequently that observations with lower SDs. The metric for determination of improvement of prediction is the PCC of the predicted EFPs and observed mouse microbiome EFPs.

### 2.4 Predict host obesity from the microbiome

A previous study (Larsen and Dai, 2015a) has suggested that microbiome community metabolome is more predictive of host dysbiosis than microbiome community structure. We use a similar approach here to predict host obesity from microbiome data.

Microbiome community metabolome was modeled using Predictive Relative Metabolic Turnover (PRMT) (Larsen et al., 2011). Briefly, PRMT is a metabolic network topology approach to quantifying the predicted relative capacity for a microbiome community to synthesize and/or catabolize specific metabolites. PRMT has been used in similar contexts in other microbiome analyses (Larsen et al., 2011; Larsen et al., 2015a; Larsen et al., 2015b).

Microbiome dataset “Obesity and Microbiome” was used for this analysis. Predictors for host diet and obesity, using either PRMT or microbiome community data, were modeled as non-linear functions, using the AI tool “Eureqa” (v 1.2) (https://www.nutonian.com/). “Eureqa” uses an evolutionary search algorithm to determine simplest mathematical equations that describe user-defined relationships in a dataset. The following functions were determined using Eureqa:

$${OBESITY}^{pop}=f\left(\text{microbiome}\;\text{community}\;\text{structure}\right)$$ (5)

$${OBESITY}^{met}=f\left(\text{predicted}\;\text{microbiome}\;\text{community}\;\text{metabolome}\right)$$ (6)

$${DIET}^{pop}=f\left(\text{microbiome}\;\text{community}\;\text{structure}\right)$$ (7)

$${DIET}^{met\;}=f\left(\text{predicted}\;\text{microbiome}\;\text{community}\;\text{metabolome}\right)$$ (8)

Where ***OBESITY*** = 1 for obese phenotype and 0 for non-obese phenotype and ***DIET*** = 1 for HF diet and 0 for LF diet. “Microbiome community structure” was all 20 bacterial taxa present in community data. For “predicted microbiome community metabolome”, the top 5% of PRMT-scored metabolites significant for Obesity, as ranked by the Fisher-score (128 metabolites) were used. All data was divided into training (80%) and testing (20%) subsets. The “Eureqa” evolutionary algorithm was allowed to run until “stability” and “convergence” were greater than 95%. Function values greater than 0.5 were considered Obese or HF, values less than 0.5 were Non-obese or LF. Accuracy of predictions were quantified as Mathews Correlation Coefficients (MCC).

### 2.5 Integrated host-microbiome model system

To demonstrate network-based models’ ability to effectively predict novel host-microbiome interactions, the collection of previously generated models were combined and used to recapitulate, *in silico*, the results of Ridura’s Microbiome Transplant Experiment.

Thethree previous models are combined such that 1) given a starting “obese” or “lean” microbiome community and a HF or LF diet, predict the changes to microbiome community structure, 2) given a predicted microbiome community structure, predict the EFP, and 3) given a predicted EFP, calculate microbiome community metabolome and host obesity. “Obese” and “Lean” microbiome community structures were taken from “Microbiome Transplant” data. An obesogenesis score, to compare results of different starting microbiomes and diets, was calculated as:

$${OBESOGENESIS}^{i}=\log_{s}\left(\frac{{OBESITY}^{met}\left(Predicted\;Metabolome\;i\right)}{Ave\_{OBESITY}^{met}\left(All\;Predicted\;Metabolomes\right)}\right)$$ (9)

Were **OBESOGENESIS** ^**i**^ is the predicted obesogenesis for microbiome *i*, **OBESITY** ^**met**^ is the function from equation (Bianchi et al., 2018), and **Ave_OBEISITY** ^**met**^ is the average **OBESITY** ^**met**^ for all predicted microbiomes. By this metric, values greater than 0 are more obese than the average host, and less than 0 are less obese/leaner than the average host.

## 3 Results

### 3.1 A network-based model is more predictive of change in microbiome community in response to diet than a non-network model

**FIGURE 1:**
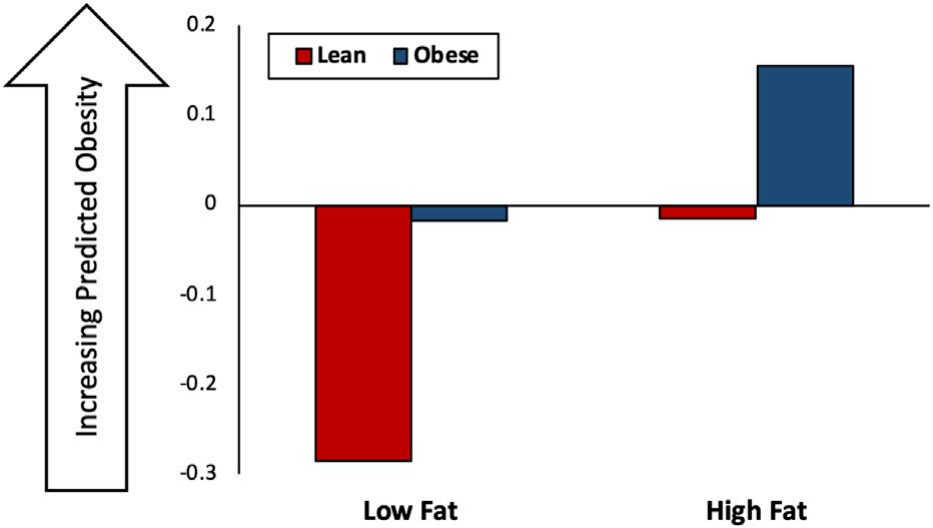
A community interaction network for mouse microbiome communities. This figure shows the result from the MAP-model. Diamonds are diet parameters, amino acids are blue, carbohydrates are yellow, fats are green, minerals are purple, and vitamins are orange. Circles are bacterial taxa and the size of bacterial node is proportionate to their average relative abundance across all analyzed microbiomes. Solid lines indicate interaction between taxa at final time point, dashed line indicate interactions between taxa at time initial and final time point.

In the generated network of microbiome community interactions constructed from the “Effect of Diet on Microbiome” dataset, 24 of the 48 (50%) possible diet parameters were found to impact the microbiome community (Figure 1). In the microbiome community interaction network, 46% of nutrient parameters are amino acids, 13% are carbohydrates, 4% are fats, 13% are minerals, and 25% are vitamins. Diet nodes are significantly enriched for vitamins, relative to the distribution of nutrient types in the total set of diet parameters (calculated as hypergeometric mean, *p*-value less than 0.05). The bacteria that have the greatest influence on population structure (i.e., have the largest out-degrees in the network) are *Parabacteroides* and *Butyrivibrio*. The bacterial nodes most regulated by other community interactions are *Desulfotomaculum*, *Ruminococcus*, *Clostridium*, and “Other”. Only *Bacteroides* and *Lactobacillus* have no parent nodes that are nutrient parameters. *Porphyromonas*, the most abundant bacteria in the mouse microbiome in this dataset, has only nutrient parameter parents and no predicted interactions with other taxa. The complete microbiome interaction network can be found in Supplementary Data Sheet S2.

**FIGURE 2:**
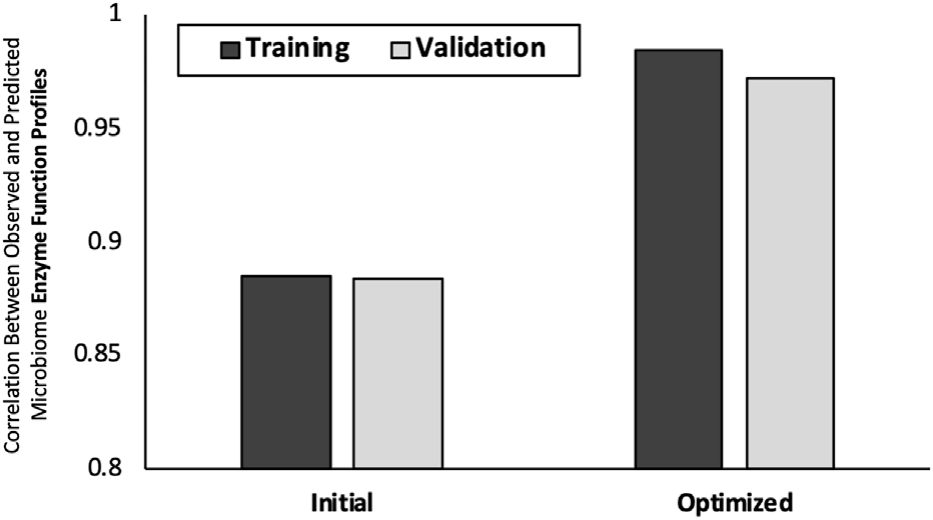
Predictions for change in microbiome in response to host diet. Y-axis is average PCC between predicted and observed mouse gut microbiome community structures for randomly generated subsets of training and validation data. On x-axis is the two modeling approaches considered: “Network-based” and “Non-network Based” models. Average results for Training and Test data subsets are shown. Error bars are +/− one standard deviation.

The average correlations between the MAP-model predicted and observed microbiome community structures are 0.92 (SD 0.20) and 0.90 (SD 0.029) for the training and test datasets, respectively. The “Non-Network” model predicts microbiome community with an average correlation of 0.56 (SD 0.027) and 0.41 (SD 0.22) compared to the observed values in the training and test datasets, respectively (Figure 2).

### 3.2 Using diverse mouse microbiome data improves predictions of microbiome enzyme function profiles

**FIGURE 3:**
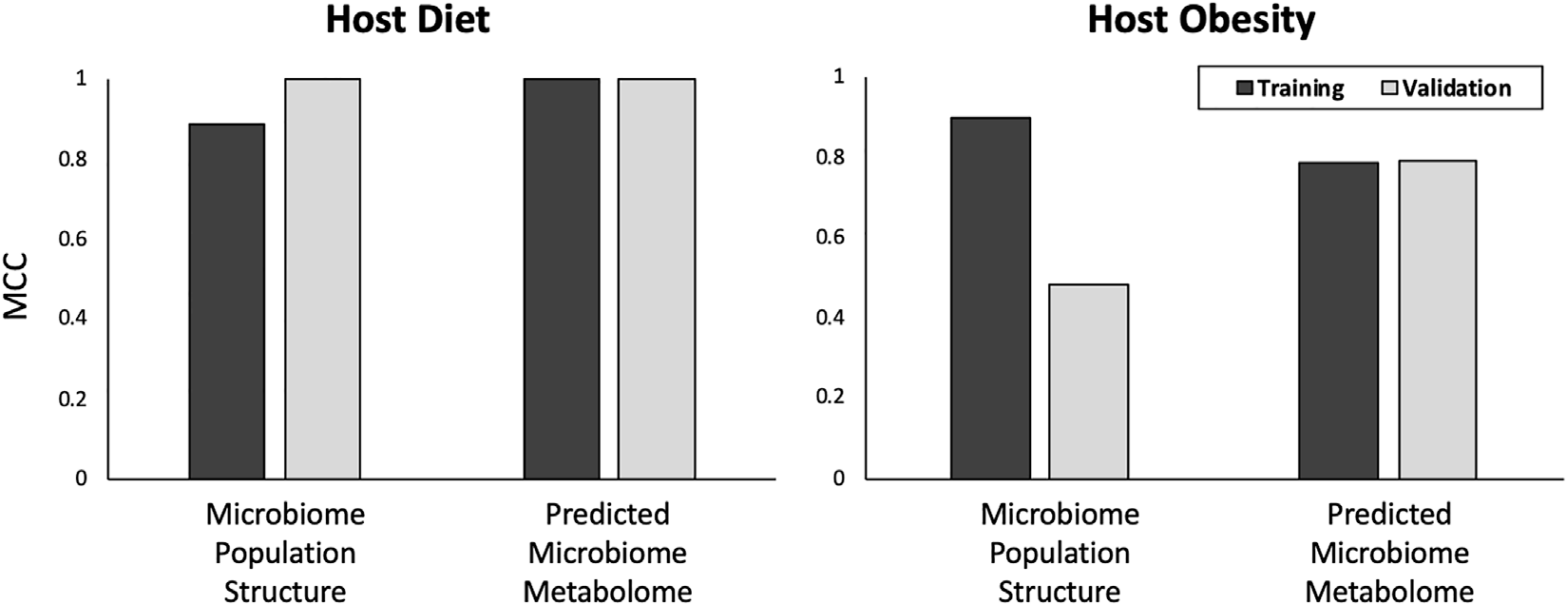
Predict microbiome enzyme function profiles from community structures. On x-axis is the two modeling approaches considered: “Initial Prediction” and “Optimized Prediction”Y-axis is the average PCC between predicted and observed mouse gut EFPs. Error bars are +/− one standard deviation.

The results of EFP-predict approach correlated with the observed EFP with PCCs of 0.88 for both training and test datasets (Figure 3). The optimized EFP predictions demonstrated a 10% increase in prediction accuracy with average PCC values of 0.96 (SD 0.005) and 0.97 (SD 0.001) for training and validation subsets respectively. Repeated runs of the Stochastic Hill Climbing approach results had percent coefficient of variation of 0.16%, indicating that the approach converges on very similar solutions in multiple runs. The complete list of observed and predicted EFPs can be found in Supplementary Data Sheet S3.

### 3.3 Microbiome community metametabolome is more predictive of host obesity than microbiome community structure

**FIGURE 4:**
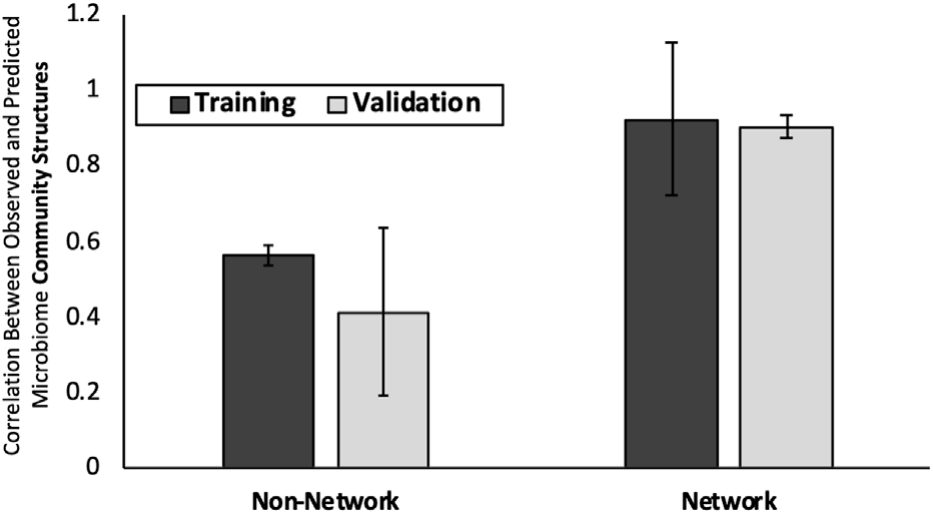
Predictions for host diet and obesity. In the top graph, results for prediction of host diet from microbiome data are shown. In the bottom graph, results for prediction of host obesity state from microbiome data are shown. Y-axis is MCC score for binary classification quality. The nature of the data used, “Microbial Community Structure” or “Microbial Community Metabolome”, to train the models are listed on the X-axis. Results are presented for both training and validation data subsets.

Both microbiome community structure and predicted microbiome community metametabolome achieved approximately equal accuracy at predicting host HF-diet phenotype with MCCs of 0.89 and 1 respectively for training subset and perfect predictions of validation subset (Figure 4). Considering host obesity however, for the test data subset, microbiome community metametabolome data generated a substantially better predictions than microbiome community structure data, with an MCC of 0.48 and 0.79 for microbiome community structure and predicted microbiome community metabolome respectively.

Tables of PRMT scores can be found in Supplementary Data Sheet S4. The models for diet and obesity can be found in Supplementary Data Sheet S5.

### 3.4 Integrated model of host-microbiome interactions correctly predicts results of microbiome transplant experiment

**FIGURE 5:**
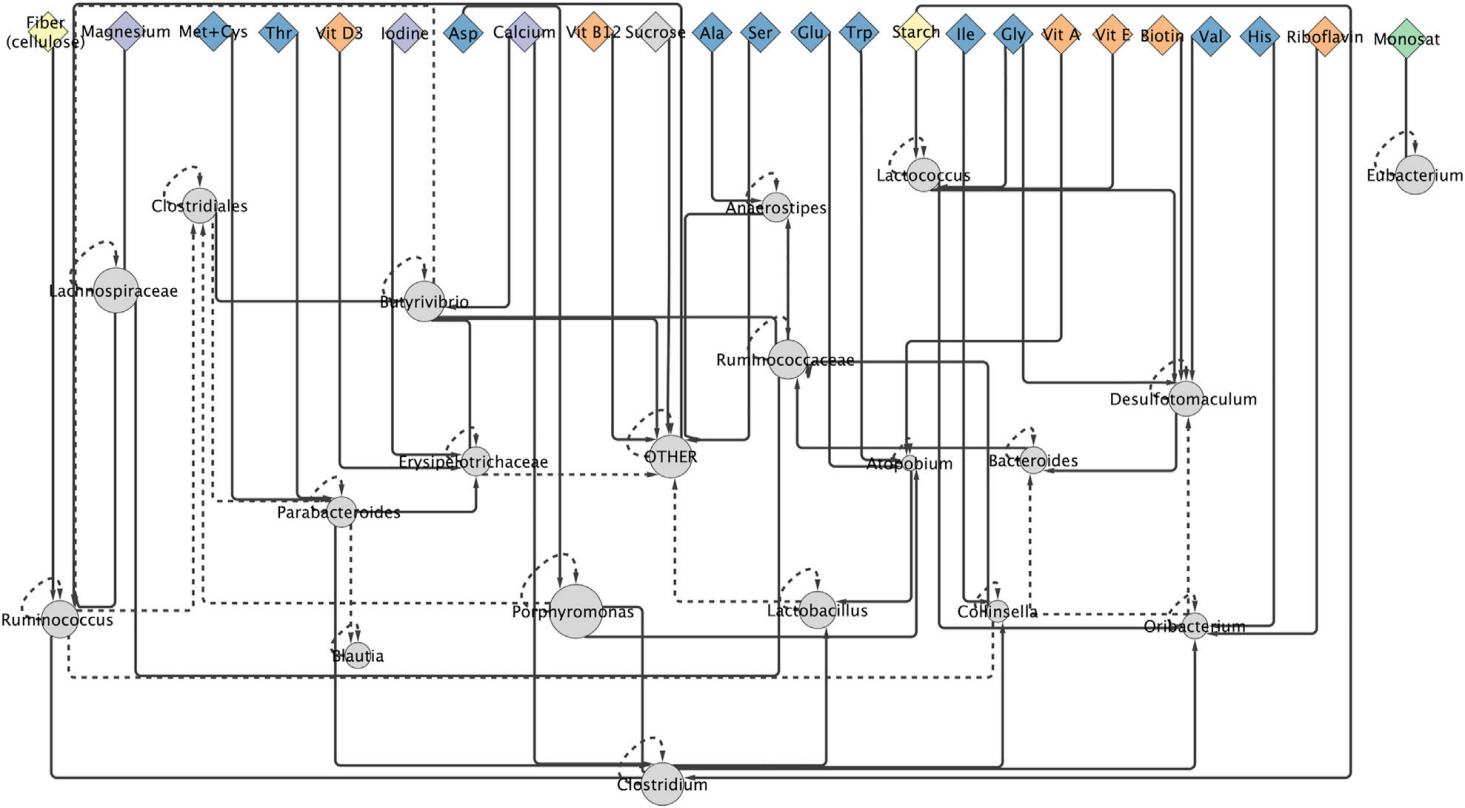
Results of *in silico* microbiome transplant experiment. The *in silico* results of the microbiome experiment, in which mice with a “Lean” or “Obese” starting microbiome are fed a Low Fat (LF) or High Fat (HF) diet. Y-axis is the predicted obesogenesis of host-microbiome-diet interactions with larger values indicated increased obesogenesis.

The results of the *in silico* microbiome transplant experiment are summarized in Figure 5. Although overall host obesogenesis increases with a HF diet, for a given diet a host with an “obese” microbiome transplant consistently have a higher predicted level of obesity than a host with a “lean” microbiome transplant.

## 4 Discussion

### 4.1 Using network-based modeling approaches improves models of obesogenesis in host-microbiome interactions

#### 4.1.1 Network-based approaches provide insight into the mechanisms of host-microbiome interaction in obesogenesis

In addition to improving the accuracy of predicting changes in MCS in response to host diet, the microbiome community interaction network (Figure 1) provides insights into the interactions between taxa and between taxa and diet parameters in the mouse gut microbiome (Figure 2). *Ruminococcus*, which is known to be associated with digestion of complex carbohydrates in the microbiome (Ze et al., 2012), is positively affected by the nutrient parameter “Fiber” in the interaction network. The only bacterial taxon associated with fat intake in the network is *Eubacterium*, which was previously shown to have an increase relative abundance in the microbiome in high fat, high sugar diets (Turnbaugh et al., 2008). The enrichment for vitamins in the set of nutrients predicted to affect the microbiome community is supported by biological observations demonstrating the important role micronutrients play in host-microbiome interactions (Biesalski, 2016; Hibberd et al., 2017; Tabatabaeizadeh et al., 2018; Waterhouse et al., 2018). The key role of vitamins in the gut microbiome community suggests a mechanism by which the microbiome could be rationally modified through manipulation of the host’s micronutrient intake.

Metabolic functions linked to metabolites associated with a HF diet are primarily associated with bacterial metabolism: amino acid metabolism, carbohydrate metabolism, biosynthesis of co-factors, and metabolism of complex ringed molecules. This is consistent with a microbial population that changes its community structure in response to new nutrient sources, which in this case is the different sugar and fat contents between a LF and HF host diet.

For metabolic functions associated with host obesity-predictive metabolites, “Glycerolipid metabolism, Fat digestion and absorption, and Vitamin digestion and absorption” are pathways associated with the host’s ability to absorb nutrients from diet rather than a bacteria’s capacity to consume them. Pathways “Neuroactive ligand-receptor interaction, Arachidonic acid metabolism, and Fc epsilon RI signaling pathway” seem to point directly to the specific molecules that mediate interactions in the gut-brain axis, interfacing the microbiome community directly with the host’s regulatory networks and perhaps even the host’s behavior. Leukotriene is directly associated with obesity (Back et al., 2014), inflammatory pathways (Busse, 1998), and response to insulin (Martinez-Clemente et al., 2011; Li et al., 2015). Phenylalanine pathways have been previously observed to be highly enriched in the microbiomes of obese hosts (Liu et al., 2017) and pyrimidine metabolism has been observed to be reduced in non-obese animals (Yang et al., 2016b). 4-Hydroxyphenylglyoxylate is an inhibitor of fatty acid oxidation that can lead to liver disease and affect the digestion of fatty acids in the gut (Keung et al., 2013). Cytidine deaminase, the enzyme responsible for deoxycytidine metabolism in the obesity-predictive metabolites, is linked to obesity-associated reduction of immune B-cell responses (Frasca et al., 2008; Frasca et al., 2016).

#### 4.1.2 The integrated multi-scale HMI-model captures properties of obesogenesis in host-microbiome interactions

Combining the individual model subsystems into an integrated, multi-scale HMI-model accurately predicts relative host obesity as a function of initial MCS and host diet conditions. As shown in the previous section, this model was used to successfully reproduce, *in silico,* the observed results of a human microbiome transplant experiment that was not used to train any model subsystems. This indicates that the host-microbiome interaction model is capable of extrapolating to biological conditions not present in its training data.

## 5 Conclusion

Multiple, independent mouse gut microbiome datasets and multiple computational modeling approaches were used to construct an integrated multi-scale model of host microbiome interactions in laboratory mouse experiments for prediction of host obesity. Models that incorporate biological networks, such as community interactions or metabolic pathways, are more predictive than similar models that do not utilize these networks. In addition, calculated biological networks in computational models identify possible molecular mechanisms of host-microbiome interactions, such as specific nutrient parameters and metabolites or metabolic pathways linked to microbiome-associated obesogenesis.

Even though the integrated HMI model is capable of successfully extrapolating to the results of the Ridura “Microbiome Transplant” experiment, the model presented here is likely too narrowly focused to be generalized much beyond the specific mouse genotypes, range of diets, and specific time-scales considered here. Additionally, while the binary identification of obesity/lean used here was a necessity derived from the need to bring together multiple, disparate published biological experiments that use different approaches for phenotypic descriptions, the label “Obesity” is a simplification of what is in actuality a complex phenotype (e.g., BMI, percent adiposity, fatty pad volume, and inflammatory response). Nonetheless, the methodology used to integrate disparate datasets into a single predictive model capable of encapsulating emergent properties of host-microbiome interactions is powerful. While, in general, accuracy of predictions, reported as Pearson’s or Mathew’s correlation coefficients, can be considered quite strong, we do not claim that alternative modeling approaches (e.g. incorporating flux balance for predicted metabolism or a sufficiently trained Deep Neural Network) might not prove at least as accurate. We do, however, propose that modeling approaches that incorporate biological networks are more predictive and insightful than a similar approaches that do not leverage biological network information. Integration of experimental datasets, as we did here, brings a risk that different experimental studies may introduce different biases that affect the prediction made by model trained on that data. While biases are certainly present in the data used here, we feel that the ability to recapitulate a biological experiment not used in the training of models indicates that, for this admittedly narrow application, those biases did not prevent us from using the model to make useful biological predictions. Microbiome community structure used in these models was mostly considered at the taxonomic level of Genera, which can be too coarse a level to accurately capture some functional interactions. For example, vitamin biosynthesis may be adequately attributed to taxa at the level of Family (Rodionov et al., 2019), but sugar utilization cannot (Iablokov et al., 2021). While the taxonomic resolution used here was suitable to predicting host obesity in this model system, other host-microbiome interaction phenotypes might require a finer level of taxonomy. A direct solution to many of these challenges could be obtained through designing and implementing specific, hypothesis driven, multi-omic host-microbiome studies specifically with the intention for use in computational model construction rather than collecting disparate, previously published datasets.

The methodology described here for the integration of multiple experimental datasets into a single, multi-scale model has broad applicability to modeling a variety of host-microbiome interaction types, will generate new insights into the interactions between microbiomes and their hosts, and will drive novel hypothesis-generated biological experiments.

## Data Availability

The original contributions presented in the study are included in the article/Supplementary Material, further inquiries can be directed to the corresponding author.
