# Supplementary material for: Modeling interaction networks between host, diet, and bacteria predicts obesogenesis in a mouse model
Source: Front Mol Biosci. 2022 Nov 15;9:1059094. doi: 10.3389/fmolb.2022.1059094 (PMC9705962; doi:10.3389/fmolb.2022.1059094)
Supplement: Supplementary file 4 [file Table4.docx]

OBESITY^met^ = 2.434 + 0.5053*MET1152 + MET1152/(0.4903 - MET904) - MET1238*MET051^2

OBESITY^pop^ = 87.68*Por + 0.4109*Bec*Eub + Cls*Col*Lact - Eub - Des*Par

DIET^met^ = 1.027 + MET070*MET063 + 8.764*MET070*MET1360 + 1.681*MET619*MET070^2*MET063^2 - 2.246*MET070*MET1018

DIET^pop^ = (8558102.7*Col*Rumi^5 - 12.59)/(3784.4 + 8547675.6*Col*Rumi^5)

Taxa are identified by the first four letters of their name. Metabolites are identifies as follows:

| MET063 | N1-(5-Phospho-alpha-D-ribosyl)-5,6-dimethylbenzimidazole |
| --- | --- |
| MET070 | Phenanthrene-4,5-dicarboxylate |
| MET1018 | Phosphonoacetaldehyde |
| MET1360 | L-Rhamnulose 1-phosphate |
| MET619 | 1D-1-Guanidino-3-amino-1,3-dideoxy-scyllo-inositol |
| MET051 | 1-Acylglycerol |
| MET1152 | alpha-Oxo-benzeneacetic acid and 4-Hydroxyphenylglyoxylate |
| MET1238 | Deoxycytidine |
| MET904 | Leukotriene D4 |
