# Supplementary material for: Modeling interaction networks between host, diet, and bacteria predicts obesogenesis in a mouse model
Source: Front Mol Biosci. 2022 Nov 15;9:1059094. doi: 10.3389/fmolb.2022.1059094 (PMC9705962; doi:10.3389/fmolb.2022.1059094)
Supplement: Supplementary file 5 [file DataSheet1.docx]

**ADDITIONAL FILES**

**Additional File 1. All collected microbiome community structures as relative abundances.** Microbiome community data structures are available as an Excel file, with a worksheet for each of the microbiomes used in this study. Each worksheet has the microbiome community structure as the relative abundance of each of the 20 selected bacterial taxa (Table 1). Each worksheet contains additional experiment specific information. Worksheet ‘Gradient’ contains microbiome community structures from “Effect of Diet on Microbiome Community Diet” (28). Additional data include ‘MG.RAST’ Identifier, the numerical ID of the experimental mouse, the % High Fat Diet condition, and a given sample name. Worksheet ‘Catalog’ contains the microbiomes from the “Metagenomic Diversity of Mouse Microbiomes” (29). Additional data include a sample name and indicates whether the sample was in the training (1) or validation (0) subsets. Worksheet ‘SV129/BL6’ includes the microbiomes from “Obesity and Microbiome” (18). Additional data include mouse genotype, and diet condition (Low Fat Chow, High Fat, or High Fat with Inhibitor). Worksheet ‘Transplant’ contains microbiomes from “Microbiome Transplant” experiment (27).

**Additional File 2. Mouse gut microbiome community interaction network.** The interaction network derived from “Effect of Diet on Microbiome Data” data, pictured in Fig. 1, is available here as a text file of pair-wise interactions. Interactions are identified as ‘time’ or ‘t-1’ for interactions associated with the final time point or initial time point respectively.

**Additional File 3. Average and standard deviation enzyme function counts by taxa for EFP-prediction.** The enzyme function count tables used in EFP-predict optimization are presented in an Excel file. Worksheet ‘ObsECCounts’ is a table of EFP from “Metagenomic Diversity of Mouse Microbiomes” dataset. Worksheet ‘AveECcounts’ is a table of average number of genes annotated with a specific EC function within the taxonomic groups used in MAP-models. ‘SDECcounts’ is a table of the standard deviation for the number of genes annotated with a specific EC function within the taxonomic groups used in MAP-models. Worksheet ‘OptimizedECcounts’ is the table of average number of genes annotated with a specific EC function within the taxonomic groups after Stochastic Hill Climbing optimization.

**Additional File 4. Models predicting host diet or host obesity from either microbiome community structure or microbiome community metabolome.**  The Eureqa-derived non-linear functions used to predict host phenotype are found here.
